# Data on publications, structural analyses, and queries used to build and utilize the AlloRep database

**DOI:** 10.1016/j.dib.2016.07.006

**Published:** 2016-07-09

**Authors:** Filipa L. Sousa, Daniel J. Parente, Jacob A. Hessman, Allen Chazelle, Sarah A. Teichmann, Liskin Swint-Kruse

**Affiliations:** a Institute of Molecular Evolution, Heinrich-Heine Universität Düsseldorf, Universitätstrasse 1, 40225 Düsseldorf, Germany; b The Department of Biochemistry and Molecular Biology, The University of Kansas Medical Center, Kansas City, KS 66160, USA; c European Molecular Biology Laboratory, European Bioinformatics Institute, Wellcome Trust Genome Campus, Hinxton, Cambridge CB10 1SD, UK; d Wellcome Trust Sanger Institute, Wellcome Trust Genome Campus, Hinxton, Cambridge CB10 1SA, UK

## Abstract

The AlloRep database (www.AlloRep.org) (Sousa et al., 2016) [1] compiles extensive sequence, mutagenesis, and structural information for the LacI/GalR family of transcription regulators. Sequence alignments are presented for >3000 proteins in 45 paralog subfamilies and as a subsampled alignment of the whole family. Phenotypic and biochemical data on almost 6000 mutants have been compiled from an exhaustive search of the literature; citations for these data are included herein. These data include information about oligomerization state, stability, DNA binding and allosteric regulation. Protein structural data for 65 proteins are presented as easily-accessible, residue-contact networks. Finally, this article includes example queries to enable the use of the AlloRep database. See the related article, “AlloRep: a repository of sequence, structural and mutagenesis data for the LacI/GalR transcription regulators” (Sousa et al., 2016) [1].

## **Specifications Table**

**Table:** 

Subject area	*Biology*
More specific subject area	*Protein biochemistry*
Type of data	*Text, figure*
How data was acquired	*Literature survey and computational calculations for LacI/GalR protein variants*
Data format	*Normalized; analyzed*
Experimental factors	*Mutational data were normalized to wild-type protein activity*
Experimental features	*For structural data, intra- and intermolecular non-covalent contacts were calculated at a 5A threshold.*
Data source location	*The University of Kansas Medical Center, Kansas City, KS*
Data accessibility	*Data is within this article and available at*www.AlloRep.org

## **Value of the data**

• The AlloRep database (www.AlloRep.org) compiles extensive sequence, mutagenesis, and structural information for the LacI/GalR family of transcription regulators.
• The AlloRep database simplifies the consolidation of non-covalent structural information with mutagenesis and sequence conservation data.
• The AlloRep database can be used to benchmark computational predictions and to design synthetic transcription repressors for biotechnology.
• The example queries contained in this article can be used to improve searches of the AlloRep database.

## 1 Data

**Fig. 1:**
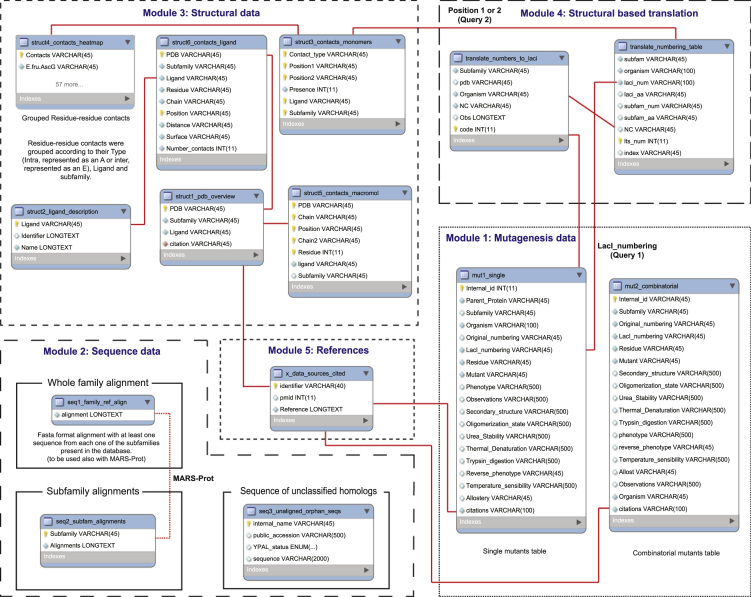
AlloRep database scheme. The five sections of the AlloRep database are contained within the dashed boxes. Each section contains one or more tables (smaller boxes with blue headings). Lines between tables indicate connections that may be accessed with SQL queries.

The AlloRep database (www.AlloRep.org) [1] compiles extensive sequence, mutagenesis, and structural information for the LacI/GalR family of transcription regulators. Phenotypic and biochemical data on almost 6000 mutants have been compiled from an exhaustive search of the literature; citations for these data are listed in this publication [2], [3], [4], [5], [6], [7], [8], [9], [10], [11], [12], [13], [14], [15], [16], [17], [18], [19], [20], [21], [22], [23], [24], [25], [26], [27], [28], [29], [30], [31], [32], [33], [34], [35], [36], [37], [38], [39], [40], [41], [42], [43], [44], [45], [46], [47], [48], [49], [50], [51], [52], [53], [54], [55], [56], [57], [58], [59], [60], [61], [62], [63], [64], [65], [66], [67], [68], [69], [70], [71], [72], [73], [74], [75], [76], [77], [78], [79], [80], [81], [82]. The data can be exported to build a local copy on the user׳s computer, but the insert and import features are disabled. New data are welcome and can be submitted to the corresponding author at lswint-kruse@kumc.edu. Here, we detail the organization of the 5 database modules and their components tables, and provide full descriptions for the contents of table columns. Fig. 1 overviews the structure of the database.

**Fig. 2:**
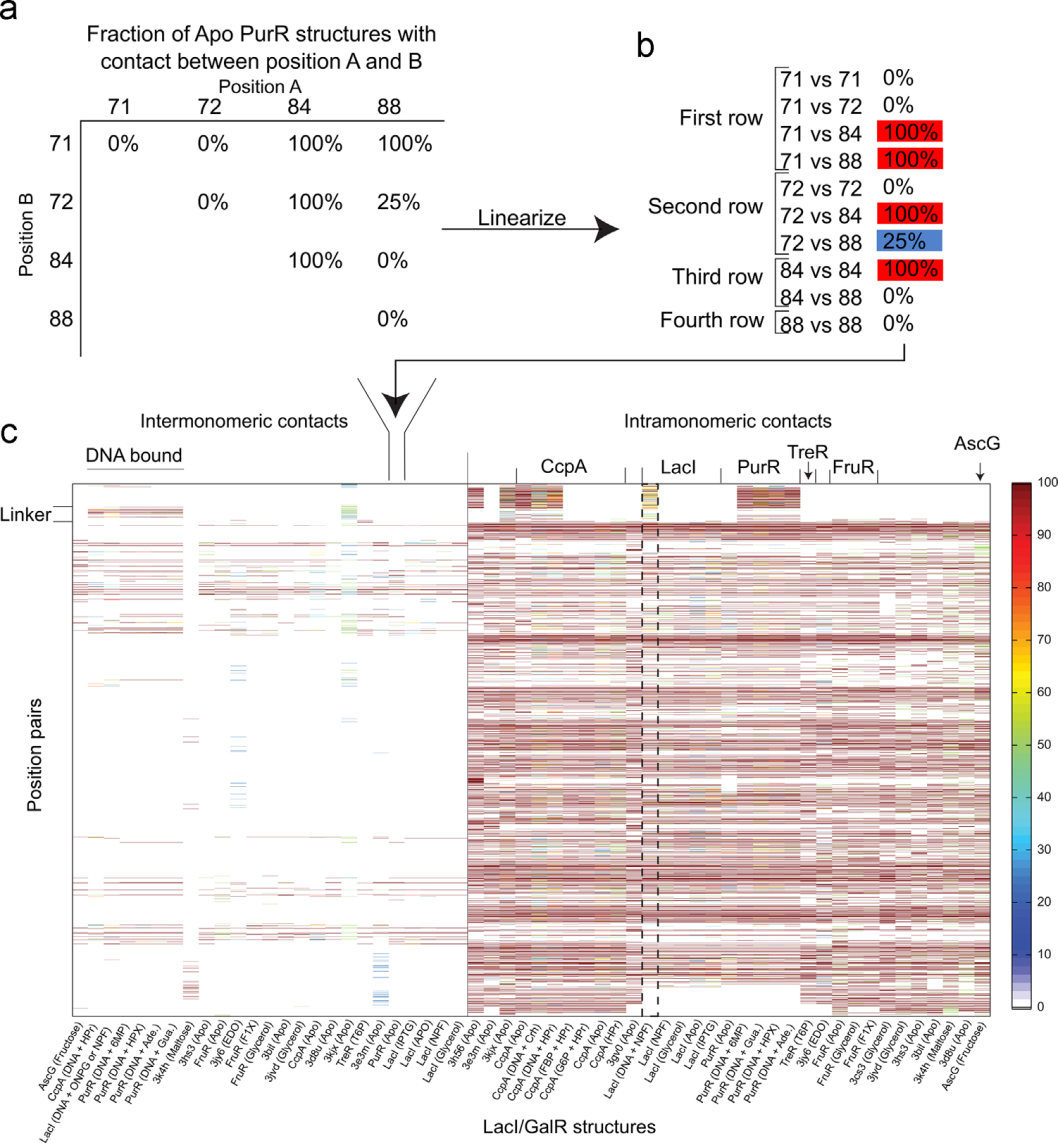
Comparisons of inter- and intra-molecular contacts among 65 structures of LacI/GalR homologs. All available structures were collected for each equivalent state of a given protein (from the same species and bound the same ligands), including the occurrences of multiple structures present in a unit cell. Inter- and intra-monomeric contacts were determined as defined in the text, and the frequency of each contact was calculated for the set of structures. If only one structure was available, the frequency was set to 100% by default. As an example, panel (a) shows an excerpt from a matrix containing information about the frequency of various contacts for all structures of *E.coli* apo-PurR. Each contact matrix was then linearized in numerical order (b) to make one column of panel (c). As a second example, the dashed box contains the composite information for all structures of LacI bound to DNA and the small molecule NPF. In panel (c), the contacts were ordered on the Y axis so that those involving the N-terminal DNA binding domain are at the top, those of the linker come next (positions 45–62 in *E. coli* LacI), followed by contacts in the regulatory domain. Each column along the X axis corresponds to the named group of equivalent structures. Bound ligands are in parentheses and ligand abbreviations can be found in the table “struct2_ligand_description”. Different colors indicate the frequency that a particular contact occurs. Inter-monomeric contacts are collected on the left of panel (c). Some structures contained monomers that could not be dimerized by symmetry operations; thus their inter-monomer contacts could not be determined. Intra-monomeric contacts are shown on the right. Once contact frequency was calculated, agnostic, hierarchical clustering was used to order the inter- and intra-monomeric contacts in panel (c). These plots show that the inter-monomer contacts (left panel) cluster according to their ligand binding state. For example, the DNA bound structures for different homologs are more similar to each other than to their respective inducer bound structures. In contrast, the intra-monomeric contacts (right panel) cluster so that the structures for each LacI/GalR subfamily are most closely related, regardless of their binding state.

We also present a protein structural comparison that was facilitated by compiling the information in the structural module. Fig. 2 shows a comparison of intra- and inter-molecular contacts from a comparative study of 65 structures available for the LacI/GalR homologs.

**Fig. 3:**
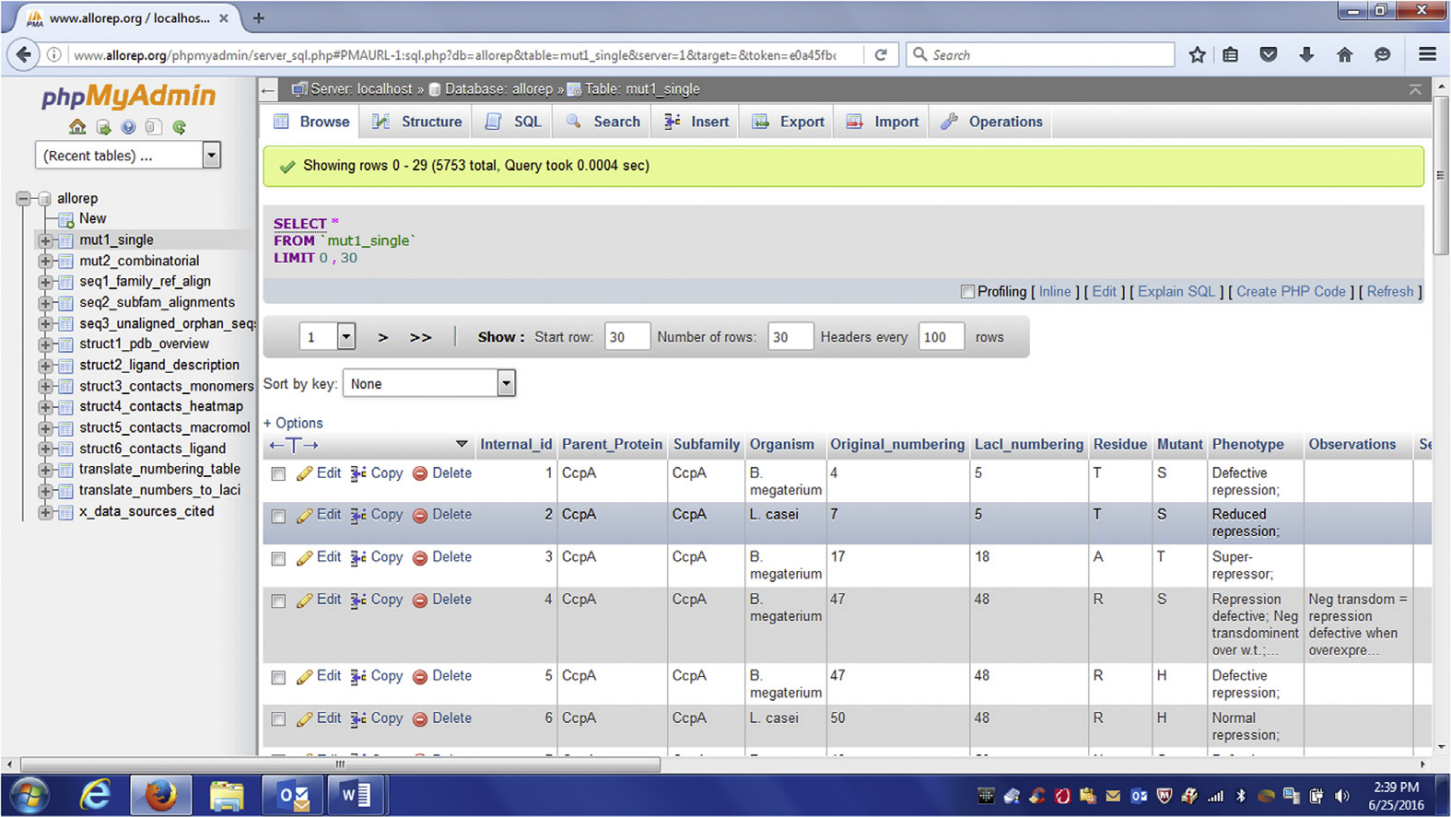
Screen shot of the AlloRep database. This screen shot was taken after entering the AlloRep database from the home webpage (www.AlloRep.org) and selecting the table “mut1_single” under the “allorep” tree that appears on the left-hand side of the window. When viewing this table under the “Browse” tab (the default option after choosing a table), individual entries can be browsed and specific features can be sorted by clicking on the column headings. For more advanced searches and filtering, the tabs near the top of the window can be used to reach the built-in search fields (“Search”) and the command-line tools (“SQL”). Example command-line queries are given in the supplement to this manuscript.

Finally, the database can be searched by selecting a table from one of the modules and using the built in search fields (search tab; Fig. 3). In addition, command line queries can be executed using the SQL tab. Example command line queries are listed in supplement to this manuscript.

## 2. Experimental design, materials and methods

### 2.1 AlloRep database overview and description of modules

The AlloRep database, freely available at www.AlloRep.org, [1] is divided into five modules (Fig. 1). Below are explanations of relevant tables and abbreviation used in each section. The tables can be browsed within the website and sorted by clicking on various column headings. In addition, example command line SQL queries are given in the supplement that can be used to link the information between the various modules.

### 2.2 Module 1: mutagenesis data

This module contains information collected from an exhaustive literature search [2], [3], [4], [5], [6], [7], [8], [9], [10], [11], [12], [13], [14], [15], [16], [17], [18], [19], [20], [21], [22], [23], [24], [25], [26], [27], [28], [29], [30], [31], [32], [33], [34], [35], [36], [37], [38], [39], [40], [41], [42], [43], [44], [45], [46], [47], [48], [49], [50], [51], [52], [53], [54], [55], [56], [57], [58], [59], [60], [61], [62], [63], [64], [65], [66], [67], [68], [69], [70], [71], [72], [73], [74], [75], [76], [77], [78], [79], [80], [81], [82]. The module entails two tables: “mut1_single” and “mut2_combinatorial”. For variants in “mut1_single”, all outcomes can be attributed to a single mutation, either by comparing the properties of a single mutation to those of the wild-type protein, or, for example, by comparing a double mutant to a variant that contains the relevant single mutation. Variants in the “mut2_combinatorial” table contain multiple mutations that have not yet been parsed into their component contributions.

Both tables contain fields for: a unique internal_id for each variant, the relevant LacI/GalR subfamily, species of origin, position number in the parent protein, position number translated to the LacI reference numbering system, one-letter codes for the original amino acid and the mutational variant, and PMIDs of the original publications. The mut1_single also contains the parent protein that provides the basis for comparison of experimental results.

In both tables, additional columns contain all available experimental information for the variant. Since experiments were carried out over several decades, in different laboratories, and sometimes under different experimental conditions (such as different buffers), the functional effects of each mutation are reported relative to the appropriate parent protein. Information regarding the effect on protein secondary structure and/or oligomerization state (where “D” stands for dimer, “T” for tetramer and “M” for monomer) are stored in columns with those names. Effects on urea stability, thermal denaturation, trypsin digestion assays, and temperature sensitivity are stored in other columns. The phenotypic and biochemical characterizations are provided in the “phenotype”, “allostery” and “reverse phenotype” columns. When possible, the relative differences are indicated with the symbols: [0] or [−−−] for total loss, [− −] for a significant decrease, [−] small decrease, [=] or ~ if comparable with wild type, [+] for small increase and [+ +] for a significant increase. Any additional information is provided in the “observation” column.

### 2.3 Module 2: sequence data

This module contains three tables with: (i) the manually-curated alignment of representative sequences for the entire LacI/GalR family (each homolog is contained in a separate row) [83]; (ii) the separate alignments for all subfamilies (each subfamily alignment is contained in one row); and (iii) a table containing unaligned “orphan” sequences (one per row) that do not match any of the current subfamilies. All data are stored in fasta format. After selecting a table of interest, it can be downloaded using the export button at the bottom of the page and selecting the desired format. Note that the output options can be customized for a better compatibility with the user׳s operating system. The subfamily alignments can be matched to the spacing of the manually-curated, whole-family alignment using the program MARS-Prot (https://github.com/djparente/MARS) [84].

### 2.4 Module 3: structural data

All available structures for LacI/GalR homologs [18], [21], [55], [57], [66], [85], [86], [87], [88], [89], [90], [91], [92], [93], [94], [95], [96], [97], [98], [99], [100], [101], [102] were retrieved from the Protein Data Bank database [103]. This module contains all the information regarding the PDB description (struct1_pdb_overview table), available ligand information (struct2_ligand_description table), and four tables with different types of contacts.

For each LacI/GalR structure, non-covalent contacts were defined when any two residues had at least one non-hydrogen atom within 5 Å of the other. Angles and other geometries were not considered. For all structures, the full set of contacts is stored in the table “struct3_contacts_monomers” where contacts were grouped according to their protein subfamily, inter- or intra-monomeric nature, and ligand. Next, for the table “struct4_contacts_heatmap”, equivalent structures (those for the same protein and liganded state) were combined to calculate the frequency of each contact pair; these values are presented in a single column for each group of equivalent structures (Fig. 2). For example, apo LacI has two structures (1lbi and 3edc) each of which contains four monomers. In two of the 8 chains (25%), LacI residues E100 and C107 are within 5 Å of each other; thus the occupancy score for this contact is 25%. For states that have only one available structure, the default value is 100%.

The table “struct5_contacts_macromol” contains information regarding the contacts between the LacI/GalR proteins and macromolecular ligands such as DNA or heteroproteins. Contacts between LacI/GalR proteins and small-molecule ligands are stored in the table “struct6_contacts_ligand table”, which also includes information on the total contact surface area and the number of contacts.

### 2.5 Module 4: translation tables

This section contains two tables – “translate_numbering_table” and “translate_numbers_to_laci” – that allow the conversion between the numbering system of *Escherichia coli* LacI and those of other LacI/GalR homologs. “Translate_numbers_to_laci” contains the necessary information for connecting both structural or mutagenesis data to the “translate_numbering_table”. The “translate_numbering_table” contains the structural alignment of all crystallographic structures as well as representative sequences for each protein subfamily that has available mutagenesis data.

Using either the PDB identifier and residue numbering as input (from tables in the structural module) or information regarding the LacI/GalR subfamily and residue numbering as input (from tables in the mutation module), the user can obtain the code to be used in the translation_numbers_to_laci and retrieve the original sequence numbering.

### 2.6 Module 5: citations

A final module comprises one table (“x_data_sources_cited”) that contains all bibliographic information and can be queried using the PMID or the citation code provided in the structural and mutagenesis tables.
