# Supplementary material for: Data on publications, structural analyses, and queries used to build and utilize the AlloRep database
Source: Data Brief. 2016 Jul 9;8:948–57. doi: 10.1016/j.dib.2016.07.006 (PMC4961497; doi:10.1016/j.dib.2016.07.006)
Supplement: Supplementary file 1 — Supplementary material [file mmc1.docx]

**Conflict of Interest:** None
