# Supplementary material for: Data on publications, structural analyses, and queries used to build and utilize the AlloRep database
Source: Data Brief. 2016 Jul 9;8:948–57. doi: 10.1016/j.dib.2016.07.006 (PMC4961497; doi:10.1016/j.dib.2016.07.006)
Supplement: Supplementary file 2 — Supplementary material [file mmc2.docx]

**Supplementary Information to: *Data on publications, structural analyses, and queries used to build and utilize the AlloRep database***

Filipa L. Sousa, Daniel J. Parente, Jacob A. Hessman, Allen Chazelle, Sarah A. Teichmann, and Liskin Swint-Kruse

**Example SQL Queries:**  These example command-line queries are also available to download as separate * .sql files. Note that the semi-colon at the end of each query is required syntax.

**Translation queries:** AlloRep can be used to translate information between the different modules of the database, using the following queries.

**Query 1:** Translate a given LacI position to analogous positions in homologs that represent other LacI/GalR subfamilies. The example given is for LacI position 35. This query also allows the user to analyze the amino acid composition conservation of particular positions using the whole-family sequence alignment comprising representative sequences from each subfamily.

SELECT * FROM translate_numbering_table WHERE (lacI_num LIKE '**35**');

**Query 2:** Retrieve the original numbering for position pairs of LacI/GalR homologs using the table “struct3_contacts_monomers”. The output will show the translation of the selected positions (in sequence numbering) as well as the amino acid composition of the selected LacI/GalR protein subfamily. Note that in the generic query 2 provided as an .sql file on the AlloRep website, the fields in bold are left empty. These should to be completed with the information regarding the residue pairs from the table “struct3_contacts_monomers”.

SELECT * FROM translate_numbering_table WHERE (lacI_num LIKE '**88**' OR lacI_num LIKE '**106**') and subfam like '**TreR**';

**Query 3**: Retrieve the original numbering from LacI/GalR proteins from the table “struct5_contacts_macromol” or “struct6_contacts_ligand”.

SELECT * FROM translate_numbering_table WHERE (lacI_num LIKE '**188**') and subfam like '**PurR**';

**Other example queries:**

**Query 4:** Conservation of intra- and inter-monomeric. non-covalent contacts among the LacI/GalR subfamilies, grouped by residue pair and type of contact.

SELECT Contact_type, Position1, Position2, count(*) FROM struct3_contacts_monomers

GROUP BY Contact_type, Position1, Position2

ORDER BY count(*) DESC;

**Query 5:** Retrieve all non-covalent residue-residue contacts for a given position. This query will allow the user to retrieve all non-covalent contacts for analogous amino acid positions, regardless of subfamily or primary sequence. This query also allows the user to retrieve all residue-residue contacts for a position selected from the mutagenesis data (“mut1_single”). Additional filters to narrow outputs are created by replacing * with the subfamily, ligand and/or contact_type of interest (inter- or intra-molecular).

SELECT * FROM struct3_contacts_monomers WHERE Position1 LIKE '**35**' or Position2 LIKE '**35**';

**Query 6-** Search for all variants with a specific phenotype (in the example, those with abolished activity).

SELECT * FROM mut1_single

WHERE phenotype LIKE '**%0%**' or phenotype LIKE '**%----%**';

**Query 7**- Retrieve all non-covalent residue-residue contacts involving positions with the same phenotype.

SELECT * FROM struct3_contacts_monomers

WHERE Position1 IN (SELECT LacI_numbering from mut1_single WHERE phenotype LIKE '**%---%**' or phenotype LIKE '**%0%**');
